# Impact of Tuning the Surface Charge Distribution on Colloidal Iron Oxide Nanoparticle Toxicity Investigated in *Caenorhabditis elegans*

**DOI:** 10.3390/nano11061551

**Published:** 2021-06-11

**Authors:** Loredana Amigoni, Lucia Salvioni, Barbara Sciandrone, Marco Giustra, Chiara Pacini, Paolo Tortora, Davide Prosperi, Miriam Colombo, Maria Elena Regonesi

**Affiliations:** Department of Biotechnologies and Biosciences, University of Milano-Bicocca, 20126 Milan, Italy; loredana.amigoni1@unimib.it (L.A.); lucia.salvioni@unimib.it (L.S.); barbara.sciandrone@unimib.it (B.S.); m.giustra2@campus.unimib.it (M.G.); chiara.pacini1991@gmail.com (C.P.); paolo.tortora@unimib.it (P.T.); davide.prosperi@unimib.it (D.P.)

**Keywords:** *Caenorhabditis elegans*, nanotoxicology, iron oxide nanoparticles, surface charge, amphiphilic polymer

## Abstract

Assessing the toxic effect in living organisms remains a major issue for the development of safe nanomedicines and exposure of researchers involved in the synthesis, handling and manipulation of nanoparticles. In this study, we demonstrate that *Caenorhabditis elegans* could represent an in vivo model alternative to superior mammalians for the collection of several physiological functionality parameters associated to both short-term and long-term effects of colloidally stable nanoparticles even in absence of microbial feeding, usually reported to be necessary to ensure appropriate intake. Contextually, we investigated the impact of surface charge on toxicity of superparamagnetic iron oxide coated with a wrapping polymeric envelop that confers them optimal colloidal stability. By finely tuning the functional group composition of this shallow polymer–obtaining totally anionic, partially pegylated, partially anionic and partially cationic, respectively–we showed that the ideal surface charge organization to optimize safety of colloidal nanoparticles is the one containing both cationic and anionic groups. Our results are in accordance with previous evidence that zwitterionic nanoparticles allow long circulation, favorable distribution in the tumor area and optimal tumor penetration and thus support the hypothesis that zwitterionic iron oxide nanoparticles could be an excellent solution for diagnostic imaging and therapeutic applications in nanooncology.

## 1. Introduction

Superparamagnetic iron oxide nanoparticles (NPs) are one of the most intensively studied colloidal NPs in biology and medicine thanks to their unique magnetic properties that make them ideal tools for several applications, including bioseparation [1], biosensing [2], their use as targeted contrast agents for magnetic resonance imaging [3,4], drug delivery [5], gene therapy [6], regenerative medicine [7] and magnetic hyperthermia treatment of cancer [8,9]. In order to obtain high-quality iron oxide NPs for biomedical applications, at least two important requirements are mandatory. Firstly, NPs should exhibit excellent and persistent magnetic properties that are mainly associated to high-purity crystalline state, which is usually achieved by synthesizing them by thermal decomposition in organic solvents at temperatures even above 300 °C [10]. The resulting NPs are coated by hydrophobic surfactants, which require be replacing or hiding by interaction with hydrophilic/amphiphilic polymers to allow their dispersion in aqueous media [11]. The presence of elevated concentrations of electrolytes and biomolecules in solution results in high surface tension of NPs, which strongly promotes their aggregation. This, in turn, is a major factor underlying their toxicity. Thus, to prevent these phenomena, the nature of NP coating is of paramount importance. In addition, the coating material is also a key element in the functionalization of the NPs with targeting molecules and drugs [11].

In general, surface functionalization with poly (ethylene glycol) (PEG) has been mostly employed to extend the NPs circulation time in living organisms [12,13]. Furthermore, although there is debate on this matter, PEG-modified iron oxide NPs are considered to be nonimmunogenic and to reduce the formation of protein corona, which make them one of the most popular polymers used for NP coating [14,15].

Currently, one of the preferred strategies to achieve colloidally stable iron oxide NPs by phase transfer relies on adding a layer of an amphiphilic polymer to NPs coated with oleate and/or oleylamine, resulting in a stabilized multidentate hydrophobic interaction by intercalation of the hydrophobic tails of the second polymer in the surfactant molecules of the as-synthesized NPs [16,17].

As above mentioned, the feasibility of engineered iron oxide NPs clinical translation is supported by a huge amount of published research. Therefore, they represent an ideal model to define general protocols for the identification of a comprehensive set of parameters for the accurate classification of risks for human health associated with the use of nanodrugs and NP-based contrast agents in biomedicine. However, much work remains to be accomplished to fully achieve this goal. NP size, size distribution, surface charge tuning, colloidal stability, batch to batch reproducibility need to be accurately controlled. In addition, standardized assays for the evaluation of both short- and long-term toxicity of NPs are strongly demanded in animal models before approval for clinical trials. However, the lack of readily available, yet reliable in vivo models for large scale screening of sets of NPs to investigate the dependence of NP toxicity from selected parameters still limits nanomedicine development toward widespread clinical practice [18].

In this context, animal models such as *Drosophila melanogaster*, *Danio rerio* and *Caenorhabditis elegans* permit scientists to conduct initial assessments on engineered nanomaterials toxicity in a simple biological system, thus facing less stringent guidelines and ethical issues with respect to research on mammals. In addition, the high compatibility showed by these organisms with high-throughput screening, including microfluidic technologies, can speed up the trail to the market [19,20]. In this work, we employed the *C. elegans* as a simple but anatomically and biologically well-defined animal model to investigate nanoparticle-bio-interactions. Despite vast evolutionary divergence, about 70% of human genes have an ortholog in the genome of *C. elegans* genome, a nematode also sharing many biological traits within human physiology, anatomy and metabolism [21]. The employment of these simple animals allows cost-effective initial biological studies on nanomaterials within common chemical laboratories [21,22]. Furthermore, their transparency, small size, prolific and short lifecycle and straightforward maintenance facilitate the assessment of the interaction between nanomaterials and a model multicellular organism [22]. The cuticle, i.e., the external envelop of the worm body, can be used as a skin model considering that its function and composition is similar to that of human skin [23,24]. Noteworthy, the *C. elegans* intestine presents a similar cellular architecture compared with higher animals in terms of cell polarity of the intestinal cells (enterocytes), including the presence of apical and basolateral domains, cell junctions, and microvilli forming the brush border [25,26,27]. Finally, the transport mechanisms of biomolecules through the biological barriers are highly conserved [28,29,30]. In fact, microscopic techniques showed that fluorescently labelled NPs are efficiently taken up by the worms during feeding, translocate to primary organs such as epithelial cells of the intestine and are secondarily sorted to organs belonging to the reproductive tract [31]. Therefore, *C. elegans* technology represents a useful tool to assess the delivery of topical and oral nanomaterials before moving to more complex model organisms [22,31]. In this work, we investigated the effect of treating *C. elegans* nematodes with 12-nm superparamagnetic iron oxide NPs (henceforth termed MYTS) coated with a common amphiphilic polymeric surfactant that allows obtaining excellent colloidal dispersion of the NPs in life-compatible aqueous solution. Besides the structural features and chemical composition of NPs, we thoroughly assessed the impact of surface charge and colloidal stability fine tuning on several physiological parameters associated with both short- and long-term toxicity of NPs.

## 2. Materials and Methods

### 2.1. NPs Synthesis and Characterization

#### 2.1.1. Chemicals and Materials

2,2′-(Ethylenedioxy)bis(ethylamine) (EDBE), Boric acid, CHCl_3_, Dodecylamine (99%), EtOH, Fluorescamine, Fluorescein isothiocyanate isomer I, MeO-PEG-NH_2_ (2 kDa), Poly(isobutylene-alt-maleic anhydride) Mw ~6000, Tetrahydrofuran (THF), were purchased from Sigma-Aldrich (Saint Louis, MO, USA). Tert-butoxycarbonyl (BOC)-EDBE was purchased from Combi-Blocks, Inc (San Diego, CA, USA). Water used in all procedures is purified by passing through MilliQ Millipore system.

#### 2.1.2. Synthesis of Poly-Maleic Acid Conjugated with Dodecylamine (PMDA)

The synthesis of PMDA was performed adding 1 g of poly(isobutene-alt-maleic anhydride) (PMA) and 0.898 g of dodecylamine to 30 mL THF anhydrous and sonicated until well dispersed. The solution was kept under stirring at 60 °C for 3 h. The volume of THF was reduced under vacuum to 10 mL, and the solution was left overnight at 60 °C. The solvent was evaporated, and the product dissolved in CHCl_3_.

#### 2.1.3. Labelling of PMDA Using FITC

A volume of 175 µL of 0.125 M Fluorescein isothiocyanate (FITC) dissolved in EtOH was added to 1 mL of 0.5 M PMDA in CHCl_3_. The solution was kept under stirring at 4 °C for 16 h in the dark. Then, the solvent was evaporated under vacuum at 40 °C and the sample dissolved in CHCl_3_ to a 0.5 M PMDA final concentration.

#### 2.1.4. Synthesis Iron Oxide Cores

Oleate-coated superparamagnetic iron oxide nanoparticles were synthesized by thermal decomposition following the procedure published elsewhere [10].

#### 2.1.5. Phase Transfer of NPs Using PMDA

Polymer coating of iron oxide NPs was done in CHCl_3_ using PMDA considering a ratio polymer/NP 150 nm^2^. Specifically, for 50 µL of 0.67 M NPs 0.67 M, 242 µL of 0.5 M PMDA was added to a total volume of 5 mL of CHCl_3_. The ratio was optimized in a previous publication [32]. The solvent was evaporated under vacuum at 40 °C, and the obtained NPs dispersed by sonication in aqueous 0.55 M Borate Buffer Saline (SBB), pH 9.0. The solution was then diluted in MilliQ and reconcentrated with Amicon Ultra centrifugal filter (100 kDa) and the PMDA-coated NPs (MYTS) content quantified by UV-Vis spectroscopy as previously described [33]. All the synthetic procedures reported here and in the following paragraphs were conducted under sterile conditions. The batch named FITC-MYTS was obtained by using PMDA-FITC instead of PMDA and following the same procedure.

#### 2.1.6. EDBE Functionalization of MYTS

A volume of 3.34 µL 0.5 M EDBE and a volume of 5.22 µL 0.1 M N-(3-Dimethylaminopropyl)-N′-ethylcarbodiimide hydrochloride (EDC) were added to 5 mg of MYTS (5 mg/mL). The mix was kept under stirring for 2 h at room temperature. Byproducts and the unconjugated EDBE were removed by Amicon Ultracentrifugal filter (100 kDa) using 0.02 M SBB, pH 8.3. The final concentration of EDBE-conjugated MYTS (MYTS-EDBE) was quantified as described for MYTS.

#### 2.1.7. EDBE Conjugation Quantification

A calibration curve was made using BOC-EDBE at different concentrations in the presence of fluorescamine. This molecule is a pro-fluorophore that develops fluorescence when reacting with a primary amine. In each well of a 96-black well plate, 200 µL of BOC-EDBE at different concentrations (0, 5, 10 and 20 µM in 20 mM SBB, pH 8.3) was added in the presence of 10 µL fluorescamine (3 mg/mL) in DMSO. Based on the calibration curve, three different concentrations of as-synthesized MYTS-EDBE were used for quantifying conjugated molecule on NPs surface. Each measurement was done in triplicate by reading the fluorescence emission at 485 nm, and the calculated conjugation efficacy (%) was 57.5 ± 1.5. Per 1 g of NPs, there are 5.21 10–5 moles of conjugated EDBE. NPs molarity was calculated considering the concentration detected by inductively coupled plasma analysis, the NPs density and size, and the molar ratio of EDBE/NPs was found around 190.

#### 2.1.8. PEGylation of MYTS 

For 5 mg of MYTS (5 mg/mL), 5 µL of 0.05 M MeO-PEG-NH_2_ (2 kDa) and 5.22 µL of 0.1 M EDC was added and the mix kept under stirring for 2 h at room temperature. The presence of by-products and the unconjugated PEG were removed by Amicon Ultracentrifugal filter (100 kDa) using 0.02 M SBB, pH 8.3. The final concentration of PEG-conjugated MYTS (MYTS-PEG) was quantified as previously mentioned.

#### 2.1.9. Synthesis of FITC-MYTS-EDBE and FITC-MYTS-PEG

NPs fluorescently labelled with FITC (FITC-MYTS-EDBE and FITC-MYTS-PEG) were obtained following the same procedure reported for EDBE and PEG functionalization, respectively, using FITC-MYTS as a starting material.

#### 2.1.10. NPs Characterization

Polymer coated NPs (MYTS) were characterized by Jeol JEM 2100Plus (Jeol, Tokyo, Japan) electron microscope (TEM) equipped with a 9 MP complementary metal oxide superconductor (CMOS) Gatan Rio9 digital camera (Gatan, Inc. Pleasanton, CA, USA). Dynamic Light Scattering (DLS) and ζ-potential measurements of MYTS, MYTS-EDBE and MYTS-PEG (0.01–0.05 mg/mL NPs, at pH 7.2) were carried out using a Malvern Zetasizer (Malvern Instruments, Malvern, UK).

### 2.2. C. elegans Strain, Maintenance and Synchronization

The nematode wild-type Bristol N2 strain provided by the *C. elegans* Genetics Centre (University of Minnesota, MN, USA) was used. Worms were grown at 20 °C on Nematode Growth Medium (NGM) plates seeded with live *Escherichia coli*, strain OP50, used for nutriment as reported [34]. To prepare age-synchronized nematodes, ten gravid adult worms were transferred in a plate with fresh NGM agar seeded with OP50 *E. coli* and allowed to lay eggs for 12 h at 20 °C. After removing the adults, plates were kept again at 20 °C for three days until the newborn worms became young adults (1-day adult worms). To rule out that the presence of *E. coli* cells might differently affect NPs uptake depending on their properties, worms were kept in axenic cultures throughout the experimentation.

### 2.3. Toxicity Evaluation of Different NPs Concentration

Sixty age-synchronized worms were placed on freshly prepared NGM plates with three different NPs concentrations (0, 10, 50 and 100 µg/mL). Every 24 h, recovered live worms were counted and transferred onto a new agar plate in the presence of the respective NPs concentration. Worms were counted as dead if they did not respond to gentle stimulation with platinum transfer pick.

### 2.4. Evaluation of C. elegans NPs Uptake

#### 2.4.1. Confocal Microscopy Analysis

Age-synchronized nematodes (100 worms/100 µL) were fed with 50 µg/mL of MYTS, MYTS-PEG or MYTS-EDBE conjugated with FITC, suspended in water and incubated for 2 h at 20 °C in agitation (70 rpm, Infors-AG CH-4103 Bottmingen agitator, Bottminger, CH). The control was incubated only with water. After treatment, worms were rinsed in PBS (20 mM potassium phosphate, pH 7.2, 0.15 M NaCl), placed in a PBS buffer drop with 5% glycerol and anesthetized with 1% sodium azide. Fluorescence was observed using a Nikon confocal microscope system A1 and processed with Nikon NIS-Elements AR. Images were captured using both a FITC filterset and a DAPI filterset in order to selectively identify the signal of FITC-NPs and distinguish it from the fluorescence emitted by lipofuscin aggregates.

#### 2.4.2. Graphite Furnace Atomic Absorption Spectroscopy (GFAAS) Analysis

Iron bioaccumulation was determined using GFAAS analysis. About 3000 1-day synchronized worms were placed on freshly prepared NGM plates and treated with 100 μg/mL of each of the three NPs (or with water as a control). After 30 h of incubation, worms were collected at 6000× *g* for 1 min at 4 °C and washed 3 times with PBS to eliminate the excess of NPs. Then, worm pellet was weighed, resuspended in 200 μL of PBS and stored at −80 °C. Before analysis, samples were broken up by sonication (1 min at 20% amplitude in ice-water bath for 3 times, followed by 10 s at 10% amplitude for 3 times) and digested for 10 min at 200 °C in microwave after addition of 4 mL HNO_3_ 65%. A calibration curve was used for the analysis. The measurements were made after dilution with water. Three independent experiments were performed for each NP.

### 2.5. Toxicity Assessment of the Different NPs in C. elegans

#### 2.5.1. Lifespan Assay

Seventy 1-day adult worms were placed in NGM plates together with 0, 50 and 100 µg/mL of unconjugated MYTS, MYTS-PEG or MYTS-EDBE and resuspended in water. To avoid generation overlapping, 40 µM 5’-fluorodeoxyuridine (FuDR) was supplemented to prevent hatching eggs. Nematodes were moved every other day to fresh plates in the presence or the absence of the respective NPs. Live animals were scored until all worms died. Three independent experiments were performed, and a representative trial is shown in the text.

#### 2.5.2. Reactive Oxygen Species Measurement

Age-synchronized animals (50 worms/100 µL) were incubated as described for the evaluation of NPs uptake with 50 and 100 µg/mL of unconjugated MYTS, MYTS-PEG or MYTS-EDBE. Control worms were incubated only with water. After 2 h treatment, worms were washed in PBS (25 mM potassium phosphate, pH 7.2, 0.15 M NaCl) to remove NPs and transferred into a 96-well plate. Then, 2,7-dichlorofluorescein diacetate (DCF-DA; Sigma-Aldrich Co., St. Louis, MO, USA) was added to the final concentration of 50 μM. DCF-DA is a permeable probe that is oxidized in the presence of reactive species forming the fluorescent product dichlorofluorescein (DCF). A microplate reader (Victor 3, PerkinElmer, Waltham, MA, USA) was employed to measure the fluorescence for 180 min (485 nm excitation, 530 nm emission). Three independent experiments were performed, and values were expressed as percentage of fluorescence intensity relative to control wells.

#### 2.5.3. Pumping Rate Assay

Age-synchronized animals were incubated as described for the evaluation of NPs uptake with 50 µg/mL of unconjugated MYTS, MYTS-PEG or MYTS-EDBE. Control worms were incubated only with water. After 2 h, worms were transferred onto NGM plates seeded with OP50 *E. coli*. The pharyngeal pumping rate was scored by counting the number of times the terminal bulb of the pharynx contracted in 1 min (pumps per minute: ppm). Measurements were carried out 2 and 24 h after release on plate. For each treatment, at least three movies for a minimum of six worms were recorded. Acquisitions were replayed at one half of the original speed using Windows Movie Maker to count the number of pumps.

#### 2.5.4. Fertility Assay

Ten gravid adult nematodes laid eggs on plate with and without 50 µg/mL of unconjugated MYTS, MYTS-PEG or MYTS-EDBE for 12 h at 20 °C. After three days at 20 °C, young adults were moved to fresh NGM plates with or without 50 µg/mL NPs. Each worm was placed singularly in one plate, and every day, at the same time point, they were transferred to a new plate until they stopped laying eggs. The number of offspring was manually counted. Each plate was examined after 24 h and 48 h of incubation at 20 °C. At both times, eggs and larvae were counted.

### 2.6. Statistical Analyses

Statistical analyses were performed by using Student’s *t*-test for all assays except for the lifespan. Data were expressed as the mean ± standard error and derived by three independents experiments. The threshold of statistical significance was set at 0.05 and 0.01. The survival curve and statistical analysis of the lifespan assay were performed with R software, version 3.3.3. *p*-values were calculated using the log-rank test, Kaplan–Meier Survival function.

## 3. Results

### 3.1. Synthesis and Characterization of MYTS, MYTS-EDBE and MYTS-PEG

**Figure 1:**
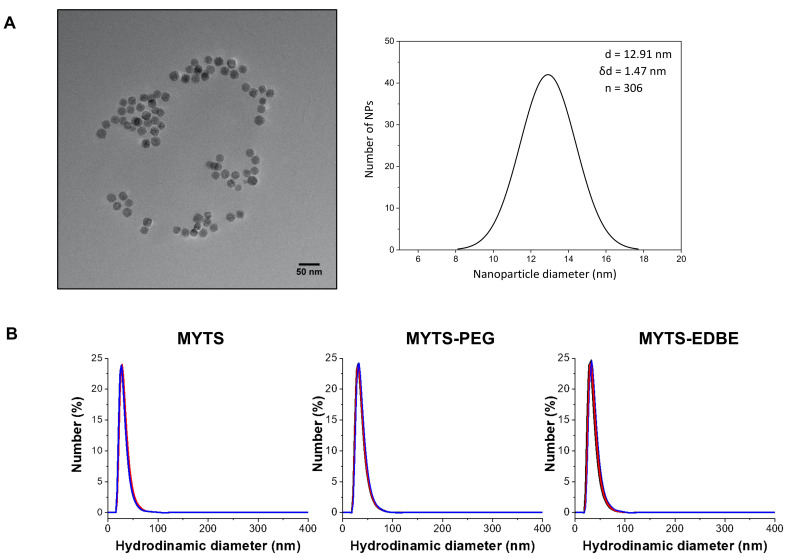
NPs characterization. (**A**) TEM Image and particle size distribution of PMDA-coated iron oxide nanoparticles; (**B**) MYTS, MYTS-PEG and MYTS-EDBE number size distribution as detected by DLS analysis (*n* = 3).

Iron oxide NPs were synthetized by thermal decomposition, which gave rise to crystalline cores of ~12 nm, as characterized by TEM analysis (Figure 1A). Then, the cores were phase transferred by adding poly-maleic acid conjugated with PMDA, an amphiphilic polymer, which is able to intercalate their hydrophobic tails in an oleic acid-covered nanosurface [35]. Notably, the synthesis of PMDA and amount required for the phase transfer were optimized in a previously published work [32]. The resulting NPs (MYTS) were then characterized by DLS and ζ-potential analyses, whereby a uniform particle dispersion with a mean hydrodynamic diameter of ~30 nm and a negative potential were detected (Table 1, Figure 1B). PDI values in the range 0.13–0.15 confirmed the uniform size of synthesized NPs and reproducibility of the measurements. MYTS were then functionalized in two ways, in both cases exploiting the large number of carboxylic groups available on NP surface. The first batch, called MYTS-EDBE, was obtained by conjugation of a homodifunctionalized EDBE onto the MYTS surface, whereas the conjugation of a heterobifunctional MeO-PEG-NH_2_ led to the synthesis of the batch here named MYTS-PEG. The reactions were conducted using similar molar amounts of reagents under the same experimental conditions. As a proof of principle, conjugation efficacy of EBDE was calculated after the detection of the free primary amine, and the molar ratio of EDBE/NPs was found around 190. DLS and ζ-potential analyses showed that neither functionalization caused substantial changes in size distribution and surface properties of the NPs (Table 1, Figure 1B). These results were expected, because the carboxylic groups displayed on the NPs surface were in large excess compared with the amount of conjugated PEG or EDBE. In summary, three batches of nanoparticles were obtained with similar physical colloidal properties but different charge distribution: indeed, compared with MYTS, MYTS-PEG and MYTS-EDBE exposed a smaller number of negatively charged groups, i.e., neutral and positively charged-groups, respectively.

### 3.2. Evaluation of the Effect of NPs Treatment in C. elegans

**Figure 2:**
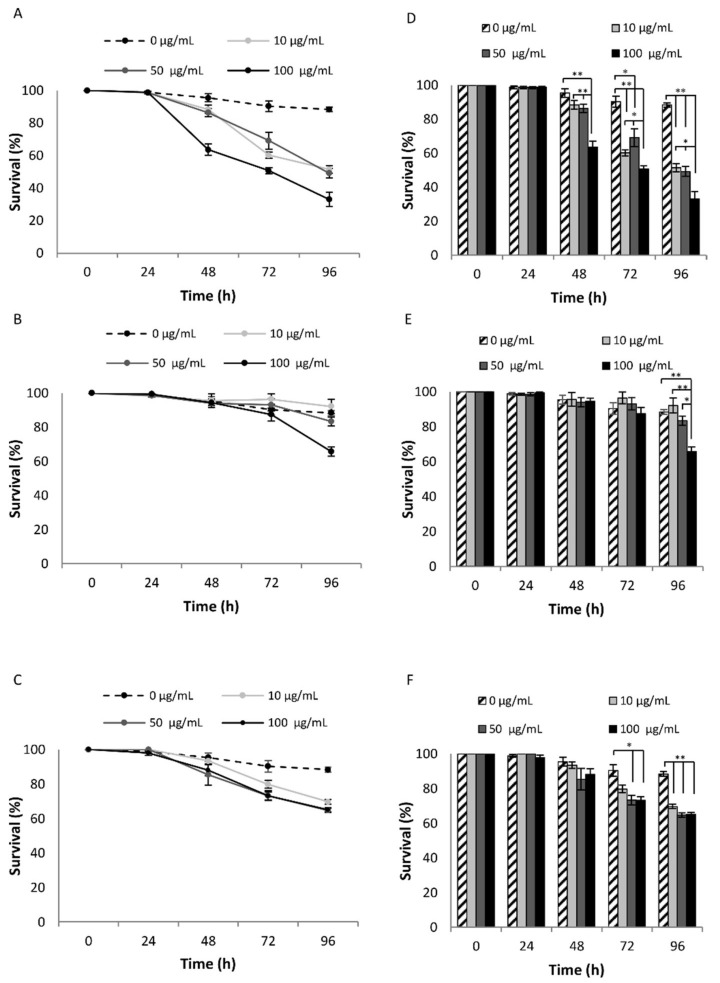
Effect of different NPs concentrations on *C. elegans* survival. Young adult animals were treated with different concentrations (0, 10, 50 or 100 µg/mL) of MYTS (**A**), MYTS-EDBE (**B**) and MYTS-PEG (**C**), respectively. Surviving and dead worms were counted at 24, 48, 72 and 96 h of treatment and expressed as percentage with respect to each 0 time. Statistical analysis of MYTS (**D**), MYTS-EDBE (**E**) and MYTS-PEG (**F**) was performed using the Student-T test. Error bars represent standard error and means derive from at least three independent experiments. * *p*-value < 0.05, ** *p*-value < 0.01.

To evaluate possible toxic effects of the three NPs on *C. elegans* viability, different concentrations (0, 10, 50, 100 µg/mL) of MYTS, MYTS-PEG or MYTS-EDBE were administered to synchronized 1-day adult worms for five days. The number of survivors were counted every day, as reported in Figure 2.

The results clearly show the toxicity of MYTS NPs (Figure 2A,D), which reduced the surviving worms already after 48 h of exposure, with a maximal reduction of about 66% (*p*-value < 0.01) at 100 µg/mL after 96 h of treatment, compared with around 35% (*p*-value < 0.01) for the two other NPs (Figure 2B,C,E,F). Noteworthy, EDBE particles gave toxicity only after 96 h of treatment at the highest concentration, underlining the significantly less toxicity of these NPs with respect to both MYTS and MYTS-PEG.

### 3.3. C. elegans Oral Ingestion of the Different NPs

**Figure 3:**
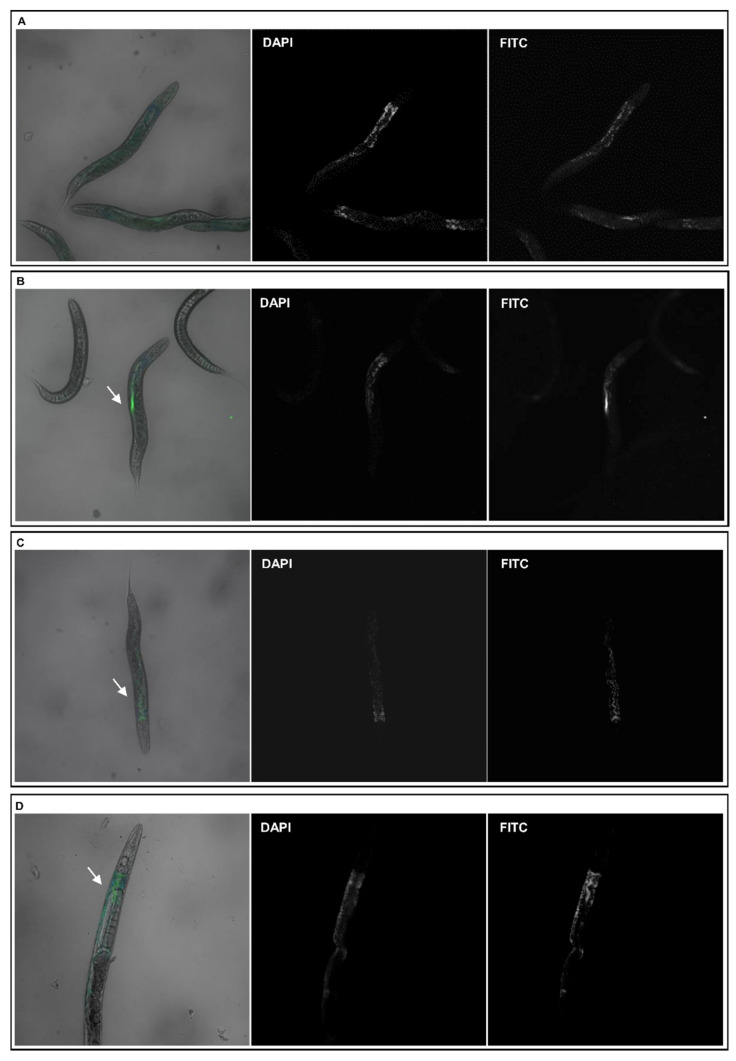
Representative confocal fluorescence micrographs of worms treated with FITC-NPs. Left column: DAPI and FITC merge; mid: DAPI; right: FITC. Worms were treated for 2 h at 20 °C under gentle agitation with the following NPs: (**A**) no addition; (**B**) MYTS, (**C**) MYTS-PEG; (**D**) MYTS-EDBE. Arrows indicate FITC-NPs fluorescence.

Since the absorption of a compound is a limiting step for its bioavailability, NPs absorption and distribution in *C. elegans* was analyzed by confocal microscopy (Figure 3). To observe the NPs directly in our model, worms were incubated for 2 h with the intermediate concentration (50 µg/mL) of NPs labeled with fluorescein isothiocyanate (FITC) dye diluted in water. Animals treated with FITC-MYTS (Figure 3B), FITC-MYTS-PEG (Figure 3C) and FITC-MYTS-EDBE (Figure 3D), presented green fluorescence distributed along the intestine, which supports the assumption that NPs uptake occurred mainly via the oral route. Control worms treated with water (Figure 3A) did not show any specific green fluorescence other than the signal pattern acquired in the DAPI channel. This confirms that the observed fluorescence in worms treated with NPs is actually due to ingested FITC-labeled NPs.

**Figure 4:**
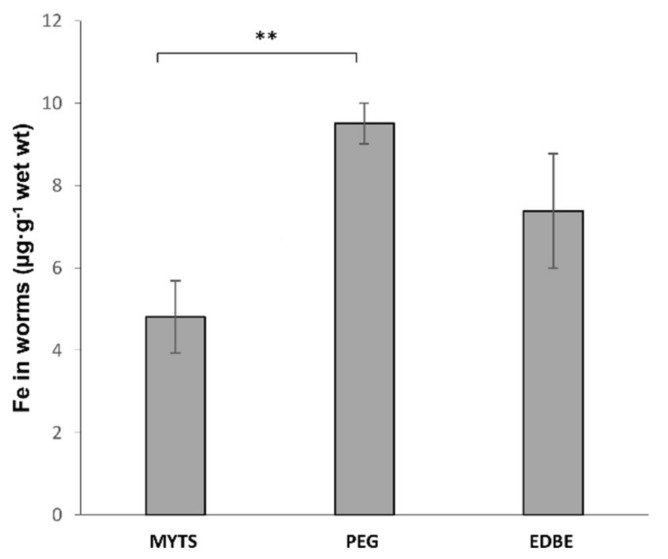
GFASS analysis of iron NPs accumulation in *C. elegans*. Synchronized worms (*n* ~3000) were treated with 100 μg/mL of unconjugated MYTS, MYTS-PEG or MYTS-EDBE and incubated for 30 h at 20 °C. Each value was obtained by subtracting the value of the control sample (incubated with no addition). Data are expressed as the mean of three independent experiments ± standard error (SE). ** *p* < 0.01 vs. MYTS was calculated by Student’s *t*-test.

Iron bioaccumulation was determined by GFAAS analysis after a 30 h incubation of worms in the presence of each of the three NPs at a 100 µg/mL concentration (Figure 4). The highest iron build-up was detected on incubation with MYTS-PEG NPs. In the case of EDBE, this was smaller but comparable, whereas it was significantly lower when MYTS NPS were administered. This clearly shows that the observed lower MYTS-PEG and MYTS-EDBE toxicity as compared to MYTS cannot be accounted for by lower NPs uptake.

### 3.4. Toxicity Assessment of the Different NPs in C. elegans

#### 3.4.1. NPs Effects on Lifespan

One-day adult worms were placed on the NGM plates in the presence of 0, 50 and 100 µg/mL of unconjugated MYTS, MYTS-PEG or MYTS-EDBE suspended in water. Plates supplemented with 40 µM FuDR were used to avoid overlapping generation. Nematodes were moved every other day to fresh plates in the presence or the absence of the respective NPs. Live animals were scored until all worms died.

**Figure 5:**
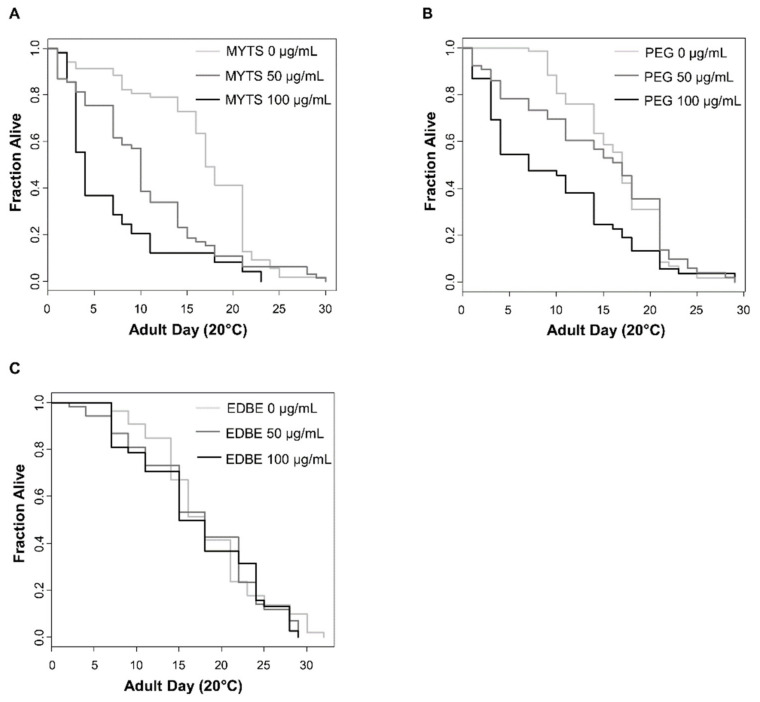
Effect of MYTS, MYTS-PEG or MYTS-EDBE on *C. elegans* lifespan. One-day synchronized worms were transferred in NGM plates with or without 50 or 100 µg/mL unconjugated MYTS (**A**), MYTS-PEG (**B**), MYTS-EDBE (**C**), respectively. The figure shows one representative experiment. Survival was scored every other day and the lifespans elaborated and compared using the Kaplan–Meier survival analysis.

The results reported in Figure 5 and Table 2 show that chronical exposure to 50 µg/mL of MYTS caused a 44% reduction in mean lifespan without any reduction in the maximum lifespan. At the highest NP concentration (100 µg/mL), the toxic effect was exacerbated, which led to a significant decrease in both mean and maximum lifespans, i.e., 81% (*p* < 0.001, Log-rank test) and 7 days, respectively (*p* < 0.001, Log-rank test) (Figure 5A, Table 2). PEG coating resulted in a considerable reduction in toxicity, as only at the highest concentration (100 µg/mL) could MYTS-PEG NPs produce significant effects, namely, a 60% reduction in the mean lifespan (*p* < 0.001, Log-rank test), without anyway affecting the maximum lifespan (Figure 5B, Table 2). Also noteworthy is that treatments with MYTS-EDBE did not shorten significantly either mean or maximum lifespan (Figure 5C, Table 2), suggesting that zwitterionic forms could eliminate long-term toxicity of nanoconjugates. Although some variability was detected among the survival profiles of the three control populations, nevertheless, the parameters characterizing these lifespan curves (i.e., mean and maximum values) are comparable, with only marginal deviations, as shown in Table 2.

#### 3.4.2. Effect of NPS on Oxidative Stress in *C. elegans*

**Figure 6:**
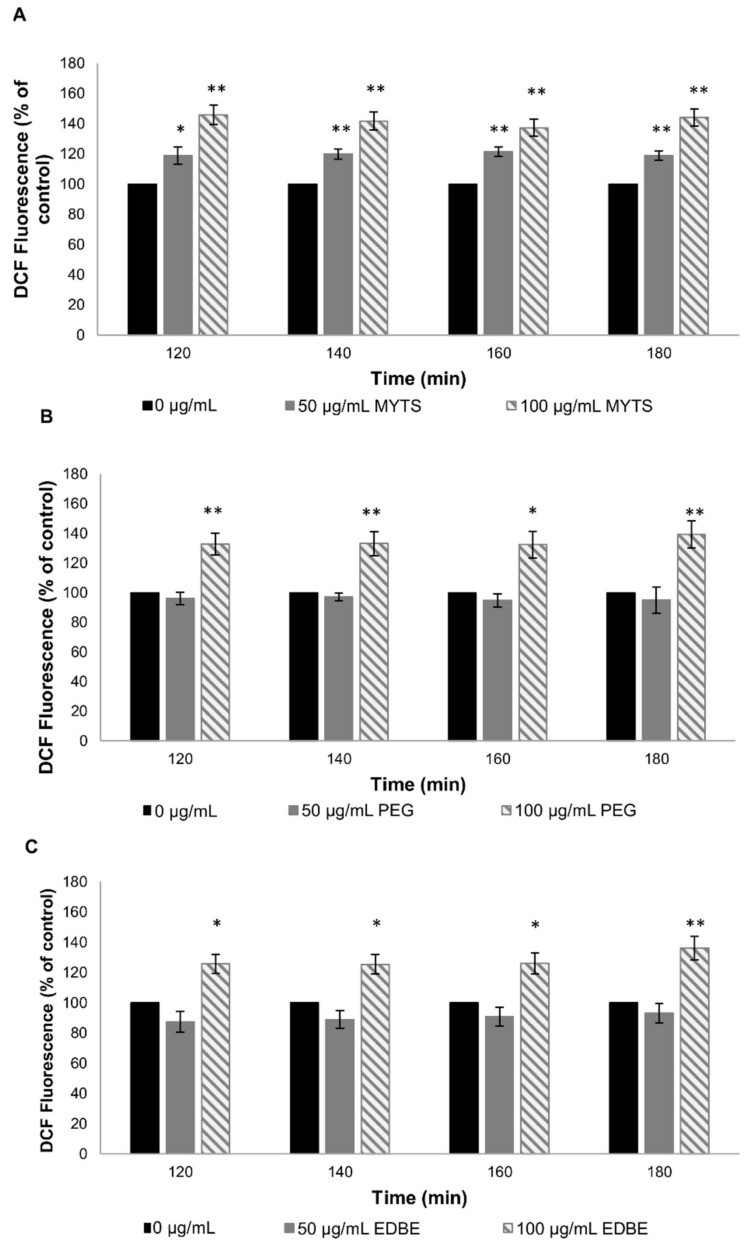
Effects of MYTS, MYTS-PEG and MYTS-EDBE on *C. elegans* ROS production. Synchronized worms (*n* = 50) were treated with or without 50 and 100 µg/mL of unconjugated MYTS (**A**), MYTS-PEG (**B**), MYTS-EDBE (**C**). NPs were diluted in water and incubated with the animals for 2 h at 20 °C in agitation (70 rpm). Control worms (0 μg/mL) were treated with water. The results are obtained from three independent experiments. Data are expressed as the mean ± standard error (SE). * *p* < 0.05, ** *p* < 0.01 vs. controls were calculated by Student’s *t*-test.

Since elevated oxidative stress plays an important role in NPs toxicity [36,37], we quantified ROS levels of the worms after treatment with 50 or 100 µg/mL of the different NPs. ROS production was determined using DCF-DA, which is converted into the oxidized DCF fluorophore in the presence of free radicals. The results reported in Figure 6 confirm that MYTS NPs display a stronger toxicity compared to the other NPs. Actually, a significant increase in DCF-DA oxidation was detected after MYTS treatment both with 50 µg/mL (22%) and 100 µg/mL (46%), while PEG and EDBE treatment induce an increase of 39% and 36% respectively only at high concentration.

#### 3.4.3. Effects of the NPs on Pumping Rate

**Figure 7:**
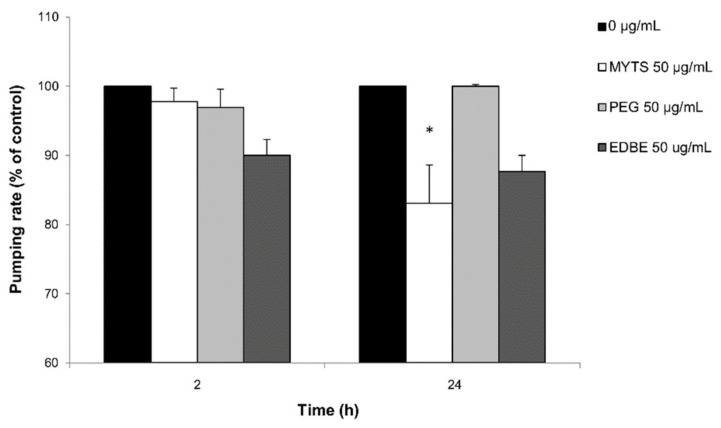
Effect of the different NPs on pharyngeal function. Young adult animals were fed with 50 µg/mL of unconjugated MYTS, MYTS-PEG or MYTS-EDBE. Control worms were fed only with water. The pharyngeal pumping was scored 2 and 24 h after plating worms on NGM plates seeded with OP50 *E. coli*. Data are expressed as the mean ± standard error (SE). * *p* < 0.05 vs. CTRL was calculated by T student test.

As a further assay aimed at assessing NPs toxicity, the rate of pharyngeal pumping was monitored. Young adult animals were fed with 50 µg/mL of MYTS, MYTS-PEG and MYTS-EDBE for 2 h before being released on NGM plates with OP50 *E. coli*. As shown in Figure 7, no significant variation of pumping rate was recorded after 2 h in plate, while a significant reduction (17% decrease with respect to the control) was scored after 24 h only for MYTS, which therefore proves to be the only NP variant endowed with a detectable toxic effect.

#### 3.4.4. Effects on Fertility and Reproduction

**Figure 8:**
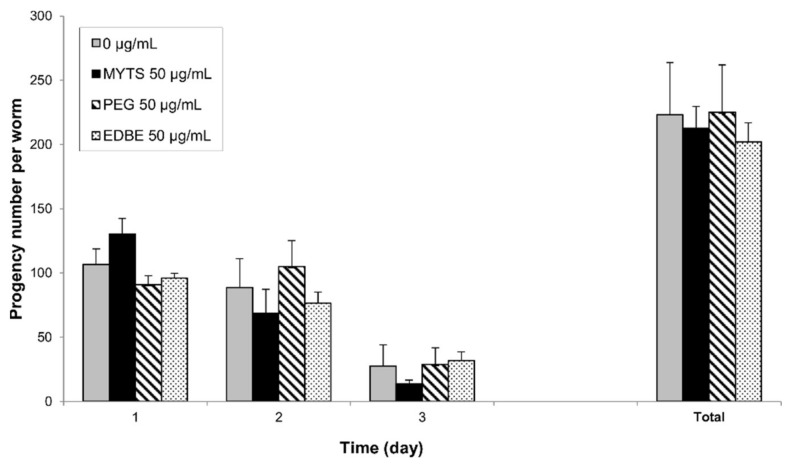
Effect of NPs on *C. elegans* reproduction. Synchronized young adult worms were placed onto NGM plates with or without 50 µg/mL of unconjugated MYTS, MYTS-PEG or MYTS-EDBE and individually transferred to a new plate each day until they stopped to lay eggs. Time-course distribution of fertility and total number were determined. Error bars represent the standard error (SE). The Student’s *t*-test did not reveal any significant difference among the treatments.

Synchronized young adult worms were placed onto NGM plates with or without 50 µg/mL MYTS, MYTS-PEG or MYTS-EDBE to evaluate progeny production of each treated group as compared to the control (Figure 8). Reproductive toxicity was assessed by calculating both the number of offspring at all ages and the size of brood. No NP produced a significant alteration of the egg-laying nor did it affect total fecundity. We conclude that NPs are comparable to the control in terms of overall fitness.

## 4. Discussion

The increasingly wider usage of superparamagnetic iron oxide NPs for research in biomedicine and for clinical applications in diagnostics and therapy is arousing concerns regarding their safety and biocompatibility. To be safely employed in biology and medicine, these NPs need to be colloidally stable in aqueous solution, often under elevated ionic strength conditions. For this purpose, colloidal NPs are generally modified on their surface to induce electronic or steric repulsion among them. While several studies have been conducted in different cell cultures to correlate NP characteristics, including size, surface charge and/or hydrophobicity, shape and chemical composition, with their toxicity, a clear and comprehensive vision of the impact of these features in vivo remains elusive [38]. This is partly due to a lack of models suitable to provide reliable information on both short-term and long-term toxicities of nanomaterials in a fast and reproducible manner, possibly on a large scale with no limitations conventionally associated with the use of animals. Here, we propose *C. elegans* nematode assays as a valuable model to investigate the impact of the above-mentioned parameters. Unlike NP safety assessment in cell cultures, *C. elegans* toxicity ranking assays offer a great deal of information from a whole animal model endowed with fully metabolically active digestive, endocrine, neuromuscular and reproductive systems [39], the reported being shown to be as predictive as rodents LD50 ranking [40]. Although still in its infancy, nematode-based models look promising in suggesting answers to some unresolved toxicity issues. In particular, the impact of surface charge on in vivo toxicity and fate of colloidal NPs has been long debated. It has been widely accepted that fully cationic NPs mostly result in high cytotoxicity in vitro, which is attributable to a combination of factors, including disruption of cell membrane integrity, proton sponge effect and production of reactive oxygen species [40] and, in vivo, to a poor biodistribution and rapid clearance by the reticuloendothelial system [41]. However, cationic NPs were suggested to improve the uptake by tumor cells, as they showed higher diffusion through the tumor mass compared with pegylated and negatively charged NPs, which is why they have not been abandoned, yet. In contrast, neutral and anionic NPs exhibited longer circulation and more favorable distribution in tumors but displayed poor cellular uptake [42]. As a result, zwitterionic NPs, combining both positive and negative surface charges, have been proposed to be the most promising option in biomedicine, especially for enhanced tumor targeting and penetration [43].

On this basis, in the present study we decided to exclude cationic NPs from the in vivo toxicity investigation and opted for the comparison among three sets of iron oxide colloidal NPs, all having net negative surface charge but differing in surface functionalization. As-synthesized NPs were coated with the negatively charged PMDA polymer (Figure 1), then conjugated or not with different molecules, generating (1) MYTS (no functionalization, fully negative); (2) MYTS-PEG (functionalized with a certain amount of neutral PEG molecules, partially negative and partially neutral); and (3) MYTS-EDBE (functionalized with a comparable amount of EDBE, partially negative and partially positive). Thus, MYTS-EDBE were designed to assess the effects of their zwitterionic nature on toxicity and uptake as compared with those displayed by fully anionic (MYTS) or pegylated (MYTS-PEG) NPs in a *C. elegans* in vivo model.

The hydrodynamic size values summarized in Table 1 are all in the range 30–35 nm, representative of non-aggregated (PDI values below 0.15), well dispersed individual nanoparticles in solution. These values did not undergo any appreciable change even after weeks (Table S1 and Figure S1 in the supporting Information). It should be noted that the degree of aggregation of colloidal dispersions depends not only on the NP characteristics, including size, surface charge and composition, but also on their concentration. For instance, recently, it was shown that using Fe NPs, the aggregation rate increases by increasing iron concentration [44]. It is difficult to quantify the impact of such effect in complex systems such as biological media; however, colloidal stability should be carefully checked during the experiments, and this unavoidable effect needs consideration when interpreting the data. In particular, the NPs used in this work proved to be stable in biological media even up to 100 mg/mL [45].

Synchronized adult worms were treated with MYTS, MYTS-PEG or MYTS-EDBE, and various life cycle parameters were assessed, including nematode short- and long-term survival after treatment; mechanism of NP entry (ingestion and/or absorption through the cuticle outer membrane); effects on *C. elegans* lifespan, pumping rates, fertility and reproduction capacity; and induction of oxidative stress. Since oxidative stress is a hallmark of NPs toxicity, ROS production is considered a highly sensitive endpoint and is used extensively for biosafety assessment of specific NPs in *C. elegans* [46,47,48].

Notably, whereas as-synthesized anionic MYTS proved to be remarkably toxic already after 48 h, only after 72 h did MYTS-PEG exhibit some toxicity at the tested concentrations, and MYTS-EDBE were non-toxic for as long as 96 h up to 50 µg/mL, showing only minor deviations from the control survival curve at 96 h (Figure 2). While previous works describing NP intake in nematode worms were conducted by pre-incubating the NPs with *E. coli* [49], here our aim was to explore the effects of the sole NPs. Thus, we first needed to investigate the way of entrance of NPs into the nematodes, whether they were preferentially absorbed through the cuticle or were instead ingested. Confocal microscopy showed that colloidally stable NPs were found in the intestine after 2 h treatment with 50 µg/mL NPs (Figure 3), confirming a preferential entry route by ingestion for all three kinds of NPs.

Lifespan is recognized as one of the toxicity endpoints to assess the effects of nanomaterials in *C. elegans* [50]. As previously reported for nanopolystyrene [51], we assumed that chronical exposure to NPs at a non-lethal concentration might cause adverse effects on lifespan, so we investigated this long-time impact on survival. While unconjugated MYTS were expected to cause reduction in both mean and maximum lifespan compared with untreated worms as they actually did (Figure 5A), MYTS-PEG exhibited only a concentration-dependent decrease in the mean lifespan at the highest concentration (Figure 5B). Furthermore, no apparent toxicity was observed after treatment with MYTS-EDBE even at 100 µg/mL after one month (Figure 5C), suggesting that the presence of both negative and positive charges conferred beneficial effects on NP safety in vivo. These results were further confirmed by analysis of ROS levels upon ingestion of NPs, which showed that only the treatment with MYTS resulted in significant ROS production (Figure 6).

Pharyngeal pumping rate is the first indicator of food intake [52]. The proper feeding rate, as well as the precise timing of pharyngeal movements is required for efficient feeding and likely for survival in nature [53]. Moreover, it is well known that feeding is inhibited by a variety of stressors [54] and pharyngeal pumping rate decreases in the presence of several known mammalian neurotoxins [39]. Here, the rate of pharyngeal pumping was monitored to evaluate the toxicity of the three kinds of NPs, showing modest effects of MYTS only at 24 h (Figure 7).

Finally, fertility assay is considered a sensitive parameter to assess treatment toxicity in *C. elegans*. In fact, it was shown that reproduction could be more sensitive to lower concentrations of chemical stressors than the concentrations affecting *C. elegans* behavior and viability [55]. In our study, no NP variety assayed significantly altered egg-laying or total fecundity (Figure 8), which confirms that such colloidally stable iron oxide NPs exert little or no such toxicity. Taken together, our results suggest that PMDA-coated iron oxide NPs (MYTS) may cause significant short-term and mild long-term toxicity, which however could be mitigated by PEGylation and completely eliminated by harnessing negatively and positively charged functional groups, thus tuning the surface charge.

In literature, several works report studies on iron and iron oxide NPs toxicity in *C. elegans* [46,47,48,56,57,58]. Pathways of iron metabolism are highly conserved between mammalians and the nematode. In particular, in the intestinal epithelium, the proteins involved in the absorption (SMF-3), storage (FTN-1, FTN-2) and export (FPN 1.1, FPN 1.2, FPN 1.3) of non-heme iron are orthologs of mammalian divalent metal transporter 1 (DMT-1), of ferritin and of ferroportin, respectively [59,60]. Largely accepted is the connection between iron dyshomeostasis-related (increase in the levels of unbound reactive iron) oxidative stress and dopaminergic and cholinergic damage in neurons [61]. Iron dyshomeostasis has a negative impact on longevity and *C. elegans* has been used as a model for this condition [62]. Detailed information about the mechanisms of iron NPs toxicity has been provided by Gonzales and coauthors [47,48]. The effect of the 6 nm superparamagnetic iron oxide NP (SPIONs) exposure in *C. elegans* was investigated by different approaches. An in vivo experimentation showed that the toxicity exerted by SPIONs cannot be accounted for by the sole release of metal ions; instead, high concentrations of NPs must exert some toxicity through nanospecific mechanisms due to their small size, high surface area-to-volume ratio or high reactivity [47]. This is confirmed by our GFASS results that clearly show no correlation between iron accumulation and toxicity after NP treatment (Figure 4). In another nanotoxicogenomic study, the molecular pathways affected by SPIONs exposure were identified [48]. In this study, it was found alteration of several key signaling transduction pathways, including Wnt, MAPK and calcium-regulated pathways, and impaired responsiveness of different biological cascades, including innate immune responses, metal detoxification and oxidative stress pathways [48]. In this complex scenario, the detected ROS increase induced by MYTS exposure strongly supports the impact of the oxidative stress in iron NPs toxicity (Figure 5 and Figure 6). However, PEG and EDBE modifications of the NPs produced only a mild reduction in MYTS-induced ROS increase, especially at the highest NP concentration (Figure 6B,C). This leads to the conclusion that the strongest reduction in lifespan, i.e., that observed after MYTS treatment, may be accounted for by additional mechanisms that deserve further investigations.

## 5. Conclusions

In the present study, we investigated the impact of modulating the surface charge of colloidally stable superparamagnetic iron oxide NPs in *C. elegans* assays. Nematodes proved to be an excellent model for studying NP toxicity also in the absence of microbial feeding, allowing researchers to acquire a large number of data sets in metabolically active in vivo experiments. By taking advantage of this animal model, we demonstrated that among the tested colloidal NPs, MYTS-EDBE were the best performing in terms of both short-term and long-term safety, suggesting that the co-presence of both negative and positive surface charges could not only improve the chances for cancer targeting, as already demonstrated in previous studies, but also confer minimal toxicity on NPs. On the horizon of this study, there is the possibility to establish a zwitterionic surface-charge paradigm for clinical translation of colloidal NPs. Further investigations in mammalian animals will be necessary to confirm the general validity of our results.

## Figures and Tables

**Table 1:** DLS and ζ-Potential analyses of MYTS, MYTS-EDBE, MYTS-PEG. The hydrodynamic diameter was obtained by number size distribution. Data represent mean ± SD of three independent measurements.

	Hydrodynamic Diameter (nm)	Polydispersity Index (PDI) *	ζ-Potential (mV)
MYTS	30.3 ± 0.9	0.152 ± 0.009	−41.2 ± 0.8
MYTS-PEG	35.1 ± 0.8	0.133 ± 0.007	−37.1 ± 3.8
MYTS-EDBE	35.0 ±1.4	0.143 ± 0.010	−35.7 ± 0.3

* Polydispersity Index (PDI) is a useful parameter to determine the size distribution of a colloidal nanoparticle dispersion in a certain medium. Conventionally, PDI values below 0.3 suggest that the quality of size distribution is good, while below 0.15 is excellent.

**Table 2:** Effect of the different NPs on longevity in *C. elegans*.

	Concentration (µg/mL)	Number of Worms	Mean Lifespan (day) ^(1)^	Maximum Lifespan (day) ^(2)^	*p*-Value ^(3)^
**MYTS**	0	70	17.0 ± 0.40	30 ± 0.33	
	50	70	9.5 ± 0.70	30 ± 0.58	1.67 × 10^−5^
	100	56	3.3 ± 0.45	23 ± 0.33	1.61 × 10^−10^
**MYTS-PEG**	0	70	16.4 ± 0.60	29 ± 0.57	
	50	65	16.0 ± 0.43	29 ± 0.33	0.823
	100	68	6.5 ± 0.65	29 ± 0.34	8.45 × 10^−5^
**MYTS-EDBE**	0	55	16.6 ± 0.62	31 ± 0.57	
	50	53	15.9 ± 0.60	29 ± 0.57	0.748
	100	42	15.3 ± 0.50	29 ± 0.33	0.45

^(1)^ Mean lifespan is defined as the age at which there is a 50% survival in the population studied. Mean ± SE was reported. ^(2)^ Maximum lifespan is the death age of the last surviving worm in each group. ^(3)^ *p*-value was calculated using the log-rank test by comparing each MYTS concentration-treated group with the respective control (0 µg/mL of MYTS).

## Data Availability

The data presented in this study are available on request from the corresponding author.

## Supplementary Materials

The following are available online at https://www.mdpi.com/article/10.3390/nano11061551/s1, Table S1: NPs stability—DLS analyses of MYTS, MYTS-EDBE, MYTS-PEG after 25 days. The hydrodynamic diameter was obtained by number size distribution. Data represent mean ± SD of three independent measurements., Figure S1: Stability over time of a representative batch of PMDA-coated iron oxide NPs (MYTS-EDBE). The hydrodynamic diameter was obtained by number size distribution. Data represent mean ± SD of three independent measurements.
