# Supplementary material for: Impact of Tuning the Surface Charge Distribution on Colloidal Iron Oxide Nanoparticle Toxicity Investigated in Caenorhabditis elegans
Source: Nanomaterials (Basel). 2021 Jun 11;11(6):1551. doi: 10.3390/nano11061551 (PMC8230852; doi:10.3390/nano11061551)
Supplement: Supplementary file 1 [file nanomaterials-11-01551-s001.zip › nanomaterials-1252603-supplementary.pdf]

*Supporting Information*

# Impact of Tuning the Surface Charge Distribution on Colloidal Iron Oxide Nanoparticle Toxicity Investigated in *Caenorhabditis elegans*

**Loredana Amigoni †, Lucia Salvioni †, Barbara Sciandrone, Marco Giustra, Chiara Pacini, Paolo Tortora, Davide Prosperi, Miriam Colombo * and Maria Elena Regonesi ***

Department of Biotechnologies and Biosciences, University of Milano-Bicocca, 20126 Milan, Italy; loredana.amigoni1@unimib.it (L.A.); lucia.salvioni@unimib.it (L.S.); barbara.sciandrone@unimib.it (B.S.); m.giustra2@campus.unimib.it (M.G.); chiara.pacini1991@gmail.com (C.P.); paolo.tortora@unimib.it (P.T.); davide.prosperi@unimib.it (D.P.)

* Correspondence: miriam.colombo@unimib.it (M.C.); mariaelena.regonesi@unimib.it (M.E.R.); Tel.: +39-02-6448-3388 (M.C.); +39-02-6448-3437 (M.E.R.)

† These authors contributed equally to this work.

**Table S1.** NPs stability - DLS analyses of MYTS, MYTS-EDBE, MYTS-PEG after 25 days. The hydrodynamic diameter was obtained by number size distribution. Data represent mean ± SD of three independent measurements.

| | Hydrodynamic diameter (nm) | Polydispersity Index (PDI) |
|---|---|---|
| MYTS | 32.4 ± 7.2 | 0.204 ± 0.013 |
| MYTS-PEG | 29.5 ± 0.5 | 0.259 ± 0.022 |
| MYTS-EDBE | 40.6 ± 7.3 | 0.228 ± 0.006 |

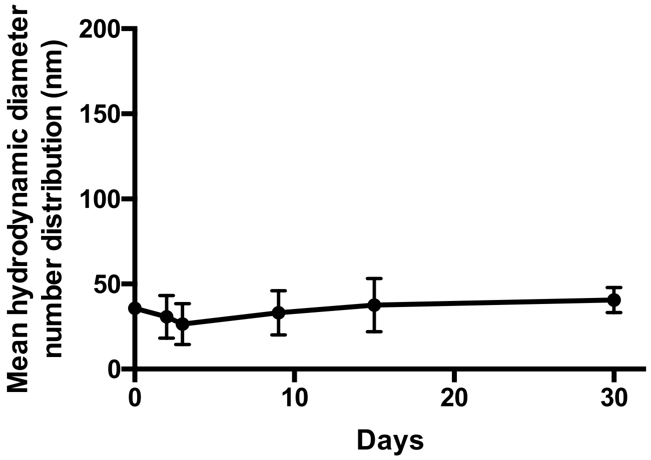


**Figure S1.** Stability over time of a representative batch of PMDA-coated iron oxide NPs (MYTS-EDBE). The hydrodynamic diameter was obtained by number size distribution. Data represent mean ± SD of three independent measurements.
